# Acupuncture Can Regulate the Peripheral Immune Cell Spectrum and Inflammatory Environment of the Vascular Dementia Rat, and Improve the Cognitive Dysfunction of the Rats

**DOI:** 10.3389/fnagi.2021.706834

**Published:** 2021-07-19

**Authors:** Pan Pan, Zhinan Ma, Zhen Zhang, Zhenzhen Ling, Yao Wang, Qiuping Liu, Xiaolin Lin, Pan Xu, Dan Yang, Hui Zhi, Runmin Wang, Xuezhu Zhang

**Affiliations:** ^1^First Teaching Hospital of Tianjin University of Traditional Chinese Medicine, Tianjin, China; ^2^National Clinical Research Center for Chinese Medicine Acupuncture and Moxibustion, Tianjin, China; ^3^Yunnan University of Traditional Chinese Medicine, Kunming, China; ^4^Weifang Traditional Chinese Hospital, Weifang, China; ^5^Department of Immune Regulation, Immunology Frontier Research Center, Osaka University, Osaka, Japan; ^6^Tianjin University of Traditional Chinese Medicine, Tianjin, China; ^7^Hangzhou Hospital of Traditional Chinese Medicine, Hangzhou, China

**Keywords:** vascular dementia, cognitive function, acupuncture, inflammation, immune cell, immunomodulatory

## Abstract

**Objective:**

The aim of this study is to analyze the effects of acupuncture on peripheral immune function, inflammation, and cognitive impairment in vascular dementia (VD) rats.

**Methods:**

In this study, 2-month-old healthy male Wistar rats (260–280 g) were assigned to the groups as follows: normal group (Gn, *n* = 10), sham-operated group (Gs, *n* = 10), and operated group (Go, *n* = 45). The Go group was established by permanent, bilateral common carotid artery occlusion (BCCAO). Two months after operation, the operated rats were screened by hidden platform trial and the rats with cognitive dysfunction were further randomly divided into impaired group (Gi), acupoint group (Ga), and non-acupoint group (Gna) with 10 rats in each group. The Ga group was given acupuncture treatment for 14 days with a rest for every 7 days. After treatment, the Morris water maze (MWM) test was performed to evaluate the spatial learning and memory abilities of rats. The lymphocyte subsets in peripheral blood and spleen of rats were measured by flow cytometry. The levels of cytokines [i.e., interleukin (IL)-1β, IL-2, IL-4, IL-10, tumor necrosis factor-α (TNF-α), and interferon-γ (INF-γ)], chemokines (i.e., macrophage inflammatory protein-2 (MIP-2)), and other inflammatory mediators (i.e., cyclooxygenase-2 (COX-2) and inducible nitric oxide synthase (iNOS)) in peripheral blood and hippocampus were measured by enzyme linked immunosorbent assay (ELISA).

**Results:**

Compared with the Gn group, the Gi rats presented long escape latencies to find the platform. After acupuncture treatment, the escape latencies of the Ga group were rescued markedly when compared with the Gi group (*P* < 0.05). The proportion of CD4 + T lymphocytes in both spleen and peripheral blood in the Ga group increased (*P* < 0.05) in comparison with the Gi group. There is an obvious reduction in IL-1β (*P* < 0.05), IL-2 (*P* < 0.05), TNF-α (*P* < 0.01), INF-γ (*P* < 0.01), MIP-2 (*P* < 0.05), and iNOS (*P* < 0.01), coming along with the increased levels of IL-4 and IL-10 (*P* < 0.01) in the Ga group when compared with the Gi group. In addition, the hippocampus proinflammatory factors IL-1β (*P* < 0.01), IL-2 (*P* < 0.01), TNF-α (*P* < 0.05), INF-γ (*P* < 0.05), MIP-2 (*P* < 0.05), iNOS (*P* < 0.01), and COX-2 decreased in the Ga group, whereas the anti-inflammatory factors IL-4 and IL-10 (*P* < 0.01) increased.

**Conclusion:**

There are abnormal immune function and peripheral inflammation in VD rats. Acupuncture can regulate the peripheral immune function and inflammation of the VD rats and can improve the cognitive dysfunction of the rats.

## Introduction

Vascular dementia (VD) is a serious cognitive dysfunction syndrome caused by cerebrovascular disease, which is the second most common type of dementia after Alzheimer’s disease (O’Brien and Thomas, 2015). The epidemiological data show that the incidence of VD is 0.98% in 71- to 79-year-old people and 4.09% in 80- to 89-year-old people (Plassman et al., 2007). However, in reality, vascular factors may have a greater impact on dementia, since some dementia-related infarcts are difficult to identify in the clinic (Smith, 2017).

In the clinic, dementia caused by ischemic brain damage is the most common type (Venkat et al., 2015). In this study, we used the bilateral common carotid artery occlusion (BCCAO) method to establish the VD rat model, which may be more stable and reliable to study the physiopathological mechanisms of cognitive impairment related to chronic cerebral ischemia (Zhang et al., 2014). The long-term decrease in cerebral blood flow caused by the ligation of bilateral common carotid arteries (BCCAs), resulting in a series of pathological changes similar to VD, such as the sparsity of brain white matter (Cho et al., 2017; Nyitrai et al., 2018), hippocampus and cortex damage (Mori et al., 2017; Nyitrai et al., 2018), increased inflammation (Hei et al., 2018; Ranjithkumar et al., 2019), microglia activation (Yuan-Cheng et al., 2018; Impellizzeri et al., 2019), oxidative stress (Zhang et al., 2017), and blood–brain barrier damage (Edrissi et al., 2016). In addition, cognitive impairment is the core symptom of dementia. Some behavioral tests also prove that the BCCAO model has poor performance in the eight-arm maze, Morris water maze (MWM), elevated T-maze, Y-maze, and object recognition test (Venkat et al., 2015), indicating that the model has a cognitive impairment such as learning and memory.

In the pathological process of ischemic brain damage and hypoxemia–hypoperfusion dementia, the chronic hypoperfusion and thromboembolism events can cause a decrease in cerebral blood flow, hypoxia, oxidative stress, and inflammatory reaction and, finally, can cause a cognitive impairment (Venkat et al., 2015). More recently, some studies have demonstrated that neuroinflammation plays a critical role in the pathophysiology of ischemic brain damage (Iadecola, 2010). According to the knowledge of the past, peripheral immune cells cannot enter the central nervous system due to the existence of the blood–brain barrier. However, more recent observations have supported that peripheral inflammation and abnormal immune function may aggravate neuroinflammation and promote neuron damage (An et al., 2014; Prinz and Priller, 2017). A typical example is that a peripheral inflammation induced by the intraperitoneal injection of lipopolysaccharide (LPS) can cause immune activation and neuroinflammation in the brain, and even cognitive impairment (Zakaria et al., 2017; Harland et al., 2020). Another example is that mice, lacking peripheral T cells, manifest cognitive dysfunction (Kipnis et al., 2004). These findings suggested that peripheral inflammation and immune dysfunction may have the adverse effects on the function of the central nervous system and may lead to the changes in cognitive function (Doyle et al., 2015; Prinz and Priller, 2017). At present, several studies have shown that the peripheral inflammation and immune function and the inflammation levels of patients with VD are abnormal (Guoping et al., 2015; Busse et al., 2017; Shang et al., 2018), while correcting the abnormalities of peripheral inflammation and immune function in patients with VD may help improve the cognitive function. Some drugs used in the treatment of Alzheimer’s disease have entered the clinical trials to modulate peripheral immune system and to improve peripheral inflammation (Dionisio-Santos et al., 2019); however, research on the peripheral immune system of VD is still rare.

Lots of evidence showed that acupuncture has immunomodulatory and anti-inflammatory effects (Zhao et al., 2015; Wan et al., 2016; Chen et al., 2017) and it has a certain curative effect on VD (Yu et al., 2006; Peng et al., 2007; Shi et al., 2012). However, the mechanism of acupuncture for VD has not been fully clarified. Existing research shows that acupuncture may improve cognitive impairment and protect neurons by enhancing antioxidant capacity (Wang et al., 2015), reducing the inflammation in the hippocampus (Fang and Sui, 2016), improving the energy metabolism level of brain tissue (Zhao et al., 2011), and promoting the angiogenesis of the hippocampus (Cai et al., 2015). There is no study to observe the regulation of acupuncture on peripheral immune function of the VD animals until now, and the relationship between acupuncture-induced peripheral immune phenotype changes and VD cognitive dysfunction is still unknown.

Therefore, we proposed a hypothesis that acupuncture treatment can improve the peripheral immune function and inflammation of VD, which may partially explain the therapeutic effect of acupuncture.

## Materials and Methods

### Experimental Animals

In this study, 2-month-old healthy male Wistar rats (260–280 g) were randomly divided into normal group (Gn, *n* = 10), sham-operated group (Gs, *n* = 10), and operated group (Go, *n* = 45). After 1 week of acclimation, the VD model was established by permanent BCCAO (i.e., 2VO). Before the operation, the rats were anesthetized intraperitoneally with 40 mg/kg of pentobarbital sodium. After the BCCAs were exposed through a neck median incision, we lightly separated them from the vagus nerve and then ligated the bilateral blood vessels with a 4–0 silk thread. After ligation, the skin incision was sutured, the iodophor was disinfected, and a 0.2 ml of gentamicin sulfate (i.e., 40 mg/ml) injection was sprayed locally to prevent wound infection. In the Gs group, the same operation was performed with the exception of arterial ligation. Throughout the surgery process, the operation was as gentle as possible to reduce the pain of the animal.

All animal experiments were performed by the guidelines of the Committee for Animal Care at Tianjin University of Traditional Chinese Medicine. Every effort had been made to minimize the number and suffering of the experimental animals.

### Acupuncture Manipulation

**FIGURE 1:**
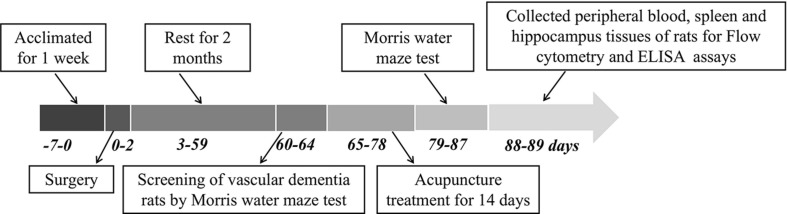
Detailed time course schema of experiments.

Two months after operation, the hidden platform trial of the MWM test was used to screen the operated rats. The screening ratio (SR) was calculated according to the formula as follows: SR = (the total mean escape latency of Go on the fifth day – the total mean escape latency of Gn on the fifth day)/the total mean escape latency of Gn on the fifth day × 100%. The SR which is higher than 20% was considered as the rats with cognitive dysfunction. The rats with cognitive dysfunction (*n* = 30) were further randomly divided into impaired group (Gi, *n* = 10), acupoint group (Ga, *n* = 10), and non-acupoint group (Gna, *n* = 10). The Ga group was given acupuncture treatment (with needles of 0.25 × 4.0 mm, Hwato, China) for 30 s. Acupuncture treatment was performed in the location of points [i.e., Tangzhong (CV17), Zhongwan (CV12), Qihai (CV6), Zusanli (ST36), and Xuehai (SP10)] as described in Table 1. The Gi rats were grasped with the same amount of time and the same extent of strength as that in the Ga group. In the Gna group, the rats were stimulated at sham sites, not related to any traditional acupuncture point, which are located at the hypochondrium on both sides of the body (i.e., 3 mm above the iliac crest) for 105 s per location (i.e., 210/2 = 105), to maintain the same stimulation techniques and time as performed in the Ga group. The treatment was continued for 14 days with a rest for every 7 days. The specific experimental steps are shown in Figure 1.

**TABLE 1:** Acupuncture points and their anatomical positions.

Points	Anatomical positions	Time(s)
Tangzhong (CV17)	On the anterior median line of the chesty, at the level of the fourth intercostal space	30
ZhongWan (CV12)	On the anterior median line of the upper abdomen, 10 mm below the xiphosternal synchondrosis	30
Qihai (CV6)	On the anterior median line of the lower abdomen, 4 mm below the umbilicus	30
Xuehai (SP10)	On the medal aspect of the thigh when the knee is flexed 4 mm above the mediosuperior border of the patella, on the bulge of the medial portion of m quadriceps femoris	30
Zusanli (ST36)	Three millimeters below head of fibula under knee joint, and 1 mm from the anterior crest of the tibia	30
Non-acupoints	On the hypochondrium, 3 mm above iliac crest	105

### Morris Water Maze Test

After treatment for 14 days, the MWM test was performed to evaluate the spatial learning and memory abilities of rats. The MWM is a circular device (i.e., 150 cm in diameter and 50 cm in height) filled with water, and the temperature of the water was maintained at 22 ± 1°C. The water surface was divided into four quadrants of equal size, and four starting positions were located in the middle of each quadrant at the edge of the tank.

The MWM test includes four stages, namely, hidden platform trial, probe trial, reversal trial, and visible platform trial. Before the hidden platform trial, a circular platform (i.e., 10 cm in diameter and 30 cm in height) was placed in the center of any quadrant and submerged 2 cm below the water surface. The rats were allowed to swim freely in the pool to familiarize themselves with the environment for two times. During the hidden platform trial, the rats were placed into the water from one of the four starting positions to find the platform. The order of the start locations was varied, and any given sequence was not repeated on the days of the acquisition phase. The rats should find the platform within 90 s, and the escape latency was recorded. If the rats failed to locate the platform within 90 s, they would be placed on the platform for 10 s to get familiar with the environment and the escape latencies of 90 s were recorded. All rats were trained two times a day for 4 days, and the average escape latency of each day was statistically analyzed.

At the end of the hidden platform trial, the probe trial was performed immediately with the platform removed. The rats were allowed to swim freely in the pool for 60 s, and the latencies of the first target-site crossover, time in the middle annulus, and the number of crossings over the former platform location was recorded.

During the reversal trial, the platform was moved to the opposite quadrant of the hidden platform trial. The rats were trained two times a day for 3 days, and the training methods were the same as the hidden platform trial.

All the rats performed the visible platform trial on the last day with the platform 2 cm above the water surface. The experimental process of the visible platform trial was the same as the probe trial.

### Flow Cytometry

After the MWM test, the rats were fasted for 12 h and then anesthetized intraperitoneally with 40 mg/kg of pentobarbital sodium. Then, the peripheral blood was collected with the ethylenediaminetetraacetic acid tube, Hebei Xinle Science & Technology Company, Hebei, China for the flow cytometry and ELISA. At the same time, the spleen and hippocampus tissues were also collected for further analysis. Erythrocytes were lysed two times with red blood cell lysis buffer (Solarbio, Beijing, China) for 15 and 10 min and then suspended two times with 1 × phosphate-buffered saline (PBS) to purify the cells. Spleens were collected as soon as possible and homogenized with the plunger of a syringe. The single-cell suspension was obtained by using a 300-μm cell filter. Then, cell suspensions were centrifuged at 300 × *g* for 10 min at room temperature and then resuspended in 3 ml of 1 × PBS. Erythrocytes were treated with red blood cell lysis buffer and washed two times with 1 × PBS. Finally, suspensions were resuspended in the appropriate volume of 1 × PBS, and the final concentration was approximately 1 × 10^6^ cells/ml and calculated by using the cell counting plate and a microscope, Olympus Corporation, Tokyo, Japan. The flow cytometry tubes were divided into negative isotype control and sample tubes with 100 μl of single-cell suspension in each tube. According to the instructions of the kit, APC-CD3 (BD Biosciences, Chicago, Illinois, United States), FITC-CD4 (BD Biosciences, Chicago, Illinois, United States), PE-CD8a (BD Biosciences, Chicago, Illinois, United States), and PE-CyTM7-CD45 (BD Biosciences, Chicago, Illinois, United States) were added into the sample tubes, and also PE-CyTM7-CD45 and T/B/NK Cell Cocktail (BD Biosciences, Chicago, Illinois, United States) were added into the isotype control tubes. After vortexed and incubated at room temperature away from the light for 20 min, each tube was added with 2 ml of 1 × PBS solution and centrifuged at 450 × *g* for 5 min. Then, the cells in the lower layer were collected, washed two times, and suspended in 1 × PBS solution, and subsequently analyzed by using flow cytometry within 1 h after staining. Lymphocytes were gated by sideward scatter and PE-CyTM7-CD45. At least 15,000 gated events were collected using FACSCalibur (BD Biosciences, United States). The data were analyzed using FlowJo X (BD Biosciences, Ashland, Oregon, United States) software. The flow cytometry gating scheme for detecting each lymphocyte subgroup is shown in this figure. First of all, according to the physical properties of lymphocytes, the lymphocyte gate is set up according to the parameters of forward scatter and sideward scatter, and then, the lymphocyte gate **(A)** is further confirmed by SSC/CD45, second, to analyze the cells in the gate, the proportion of T cells (CD3 + cells) is determined by CD3/CD45, the proportion of B cells (CD3-CD45RA + cells) is determined by CD3/CD45RA, and the proportion of NK cells (CD3-CD161a + cells) is determined by CD3/CD161a **(B)**, and finally, the proportion of CD3 + CD4 + T cells (CD3 + CD4 + cells) was determined by CD3/CD4, and the proportion of CD3 + CD8 + T cells (CD3 + CD8 + cells) was determined by CD3/CD8 **(C)**.

### ELISA

The peripheral blood of rats (*n* = 5) was centrifuged at 1,500 × *g* for 10 min at 4°C. The serum was separated and stored at −80°C until use. The hippocampus was homogenized with 1 × PBS and centrifuged at 3,000 × *g* for 10 min at 4°C, and then, the supernatant was stored at −80°C for further analysis. According to the protocols of the ELISA kit (IBL International, Germany), the levels of interleukin IL-1β, IL-2, IL-4, IL-10, TNF-α, INF-γ, MIP-2, COX-2, and iNOS were detected.

### Statistical Analysis

All the data were processed with the SPSS (IBM Corporation, Chicago, Illinois, United States) 17.0 statistical software. The data were expressed as mean ± SEM. The data of the hidden platform trial and reversal trial were analyzed by the two-way repeated-measure ANOVA with the least significant difference (LSD). The comparison between groups was performed using the one-way ANOVA with LSD. *P* < 0.05 was considered statistically significant.

## Results

### Acupuncture Ameliorated the Cognitive Disabilities of VD Rats

**FIGURE 2:**
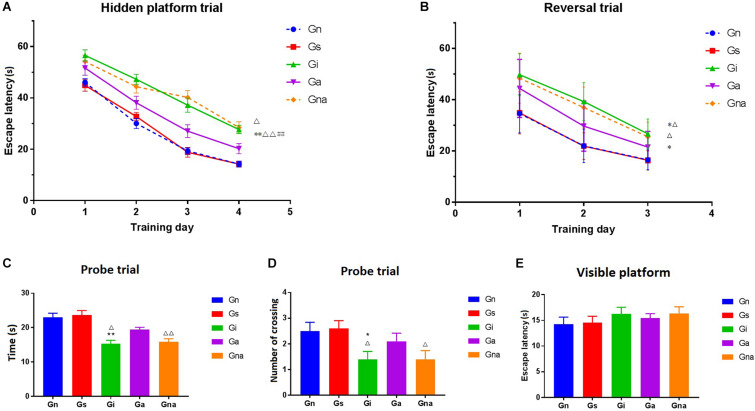
Acupuncture improved the spatial learning and memory ability of VD mice in the Morris water maze. **(A)** Escape latency of each group in the hidden platform trial. **(B)** Escape latency of each group in the reversal trial. **(C)** Time spent in the former platform quadrant in the probe trial. **(D)** The number of crossing over the former platform location in the probe trial. **(E)** Escape latency of each group in the visible platform. The data are expressed as the mean ± SEM (*n* = 10). *P < 0.05, **P < 0.01, vs. Gn group; ^#^*P* < 0.05, ^##^*P* < 0.01, vs. Gs group; ^△^*P* < 0.05, ^△△^*P* < 0.01, vs. Ga group. Gn, normal group; Gs, sham-operated group; Gi, impaired group; Ga, acupoint group; Gna, non-acupoint group.

As shown in Figure 2A, there were no significant differences in escape latency between the Gn and Gs groups (*P* > 0.05), suggesting that the operation did not affect the learning and memory ability of rats. Compared with the Gn group, the operated rats presented long escape latencies to find the platform (*P* < 0.01), indicating that the learning and cognitive function of operated rats was impaired. After acupuncture treatment, the escape latencies of the Ga group were rescued markedly when compared with the Gi group (*P* < 0.01). During the whole training process, the escape latency of the Gna group was higher than that of the Ga group (*P* < 0.05).

In the probe trial, both the time spent in the former platform quadrant and the number of crossing over the former platform location are showed in Figures 2C,D.

No obvious differences were found between the Gn and Gs groups (*P* > 0.05). The time of staying in the quadrant of the original platform of the Gi group was significantly shorter than that of the Gn group (*P* < 0.01), and the times of crossing the original platform were also less than that of the Gn group (*P* < 0.05). The retention time of the former platform of the Ga group was much longer than that of the Gi group (*P* < 0.05). Similar results were obtained from the former platform crossings between the two groups. There were no significant differences between the Gna and Gi groups (*P* > 0.05).

Figure 2B shows the time of finding the hidden platform (i.e., in a different quadrant than the one used in the original platform). The results showed that the time of searching for the hidden platform in the Gi and Gna groups was significantly longer than that in the Ga, Gn, and Gs groups (*P* < 0.05).

In the visible platform trial, there were no significant differences in escape latency among the five groups (*P* > 0.05) (Figure 2E), indicating that the above experiments were not affected by the difference in motivation and motor skills and could objectively reflect the cognitive function of rats.

### Acupuncture Can Improve the Peripheral Immune Function of VD Rats

**FIGURE 3:**
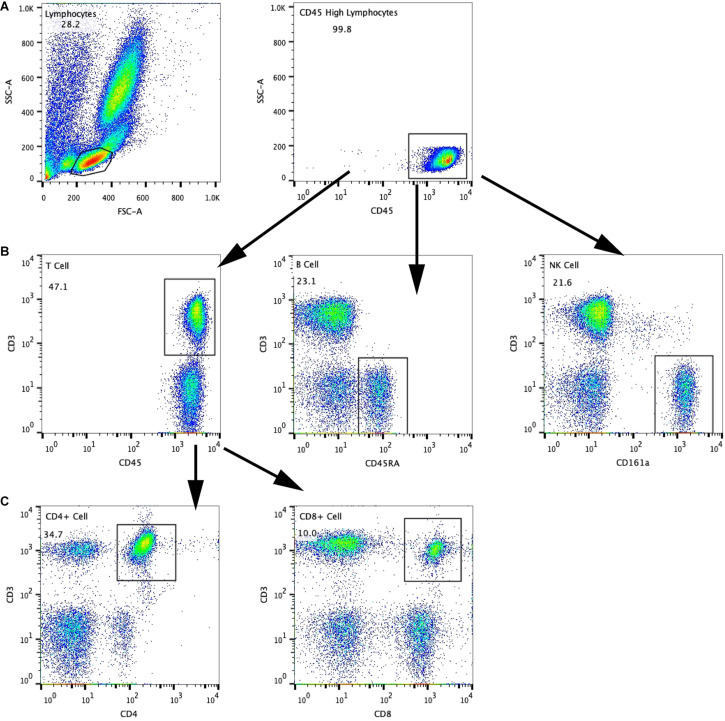
The flow cytometry gating scheme for detecting each lymphocyte subgroup is shown in this figure. First of all, according to the physical properties of lymphocytes, the lymphocyte gate is set up according to the parameters of forward scatter and sideward scatter, and then, the lymphocyte gate **(A)** is further confirmed by SSC/CD45, second, to analyze the cells in the gate, the proportion of T cells (CD3 + cells) is determined by CD3/CD45, the proportion of B cells (CD3-CD45RA + cells) is determined by CD3/CD45RA, and the proportion of NK cells (CD3-CD161a + cells) is determined by CD3/CD161a **(B)**, and finally, the proportion of CD3 + CD4 + T cells (CD3 + CD4 + cells) was determined by CD3/CD4, andthe proportion of CD3 + CD8 + T cells (CD3 + CD8 + cells) was determined by CD3/CD8 **(C)**.

**FIGURE 4:**
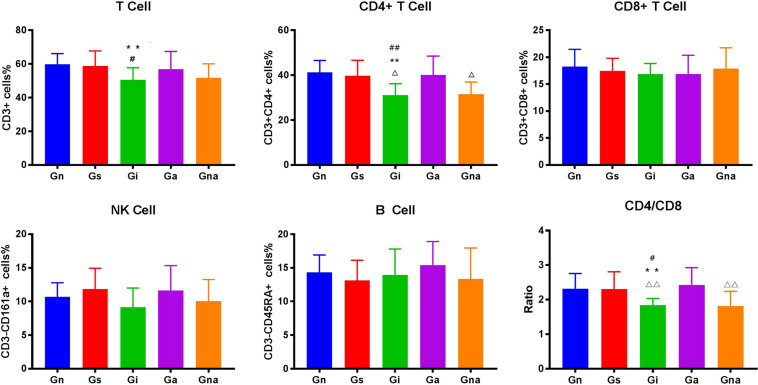
Effect of acupuncture on peripheral blood lymphocyte subsets. The data are expressed as the mean ± SEM (*n* = 10). **P < 0.01, vs. Gn group; ^#^*P* < 0.05, ^##^*P* < 0.01, vs. Gs group; ^△^*P* < 0.05, ^△△^*P* < 0.01, vs. Ga group. Gn, normal group; Gs, sham-operated group; Gi, impaired group; Ga, acupoint group; Gna, non-acupoint group.

**FIGURE 5:**
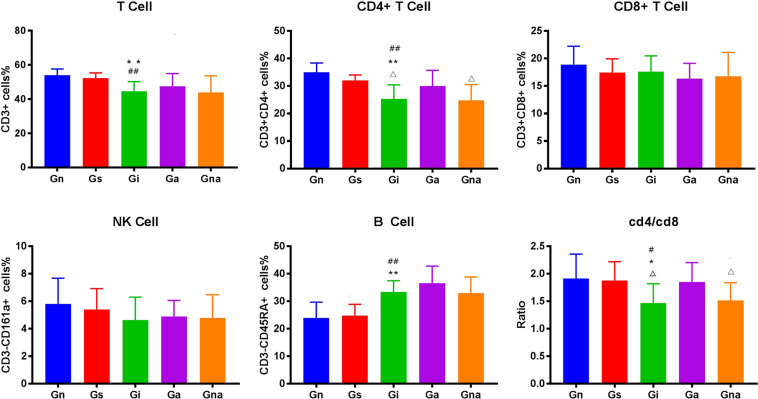
Effect of acupuncture on splenic lymphocyte subsets. The data are expressed as the mean ± SEM (*n* = 10). *P < 0.05, **P < 0.01, vs. Gn group; ^#^*P* < 0.05, ^##^*P* < 0.01, vs. Gs group; ^△^*P* < 0.05, ^△△^*P* < 0.01, vs. Ga group. Gn, normal group; Gs, sham-operated group; Gi, impaired group; Ga, acupoint group; Gna, non-acupoint group.

We studied whether acupuncture affected the proportion of lymphocyte subsets in peripheral blood and spleen. The flow cytometry gating scheme for detecting each lymphocyte subgroup is shown in Figure 3. The flow cytometric analysis showed that there were no differences in the proportion of lymphocytes in peripheral blood and spleen between the Gs and Gn groups (*P* > 0.05), suggesting that operation did not affect the proportion of lymphocyte subsets in rats. As shown in Figures 4, 5, the proportion of T lymphocytes and CD4 + T lymphocytes in peripheral blood and spleen of the Gi group was significantly lower than that of the Gn and Gs groups (*P* < 0.05), while the proportion of B lymphocytes in the spleen of the Gi group was significantly higher than that of the Gn and Gs groups (*P* < 0.01), indicating that the peripheral immune function of VD rats was abnormal.

Besides, the proportion of CD4 + T lymphocytes in both spleen and peripheral blood in the Ga group increased (*P* < 0.05) compared with the Gi group. The proportion of CD4 + T lymphocytes in both spleen and peripheral blood in the Ga group was lower than that in the Gn group, and there were no significant differences between the two groups, but the proportion of CD4 + T lymphocytes in the Ga group showed an upward trend. There were no significant differences between the Gs and Gna groups (*P* > 0.05), suggesting that the acupuncture points were of therapeutic specificity.

### Acupuncture Regulates the Expression of Peripheral Inflammatory Factors in VD Rats

**FIGURE 6:**
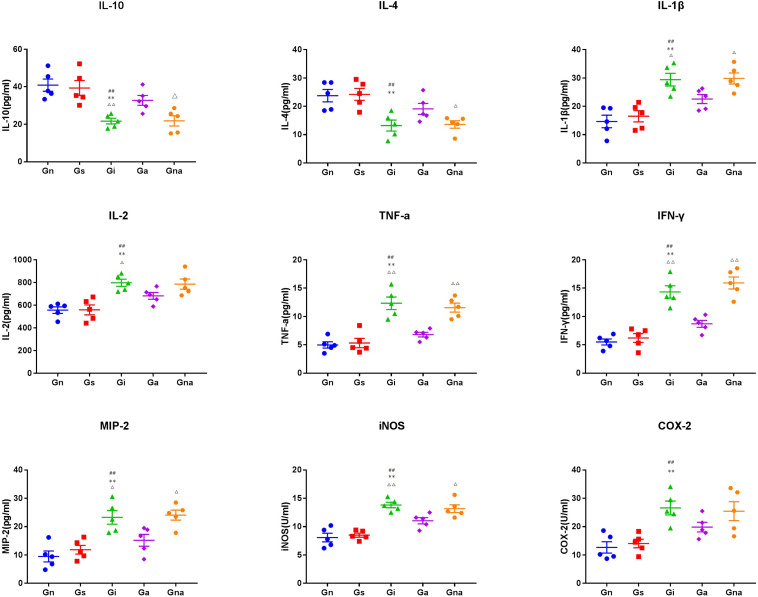
Effect of acupuncture on the levels of related inflammatory mediators in serum. The data are expressed as the mean ± SEM (*n* = 5). **P < 0.01, vs. Gn group; ^#^*P* < 0.05, ^##^*P* < 0.01, vs. Gs group; ^△^*P* < 0.05, ^△△^*P* < 0.01, vs. Ga group. Gn, normal group; Gs, sham-operated group; Gi, impaired group; Ga, acupoint group; Gna, non-acupoint group.

To verify the peripheral inflammatory level of VD rats and the effect of acupuncture on them, the serum cytokines (i.e., IL-1β, IL-2, IL-4, IL-10, TNF-α, and INF-γ) and other inflammatory mediators (i.e., COX-2 and iNOS) of each group were detected by ELISA (Figure 6). There were no significant differences in serum cytokines and inflammatory mediators between the Gs and Gn groups (*P* > 0.05), indicating that operation did not affect the serum inflammatory level in rats. As shown in Figure 6, the cytokines of IL-1β, IL-2, TNF-α, INF-γ, and MIP-2 and the inflammatory mediators of COX-2 and iNOS in the serum of the Gi group increased remarkably, while IL-4 and IL-10 decreased, which showed significant differences when compared with Gn and Gs groups (all *P* < 0.01). All the results prompted that VD rats have a higher level of inflammation. After acupuncture treatment, there is a significant reduction in IL-1β (*P* < 0.05), IL-2 (*P* < 0.05), TNF-α (*P* < 0.01), INF-γ (*P* < 0.01), MIP-2 (*P* < 0.05), and iNOS (*P* < 0.01), coming along with the increased levels of IL-4 and IL-10 (*P* < 0.01) in the Ga group compared with the Gi group. To sum up, acupuncture treatment has the potential to decrease the level of serum inflammatory factors and to relieve the inflammatory reaction in VD rats. Also, due to the specificity of acupuncture points, there were no significant differences between the Gi and Gna groups (*P* > 0.05).

### Acupuncture Can Reduce Inflammatory Factors in the Hippocampus of VD Rats

**FIGURE 7:**
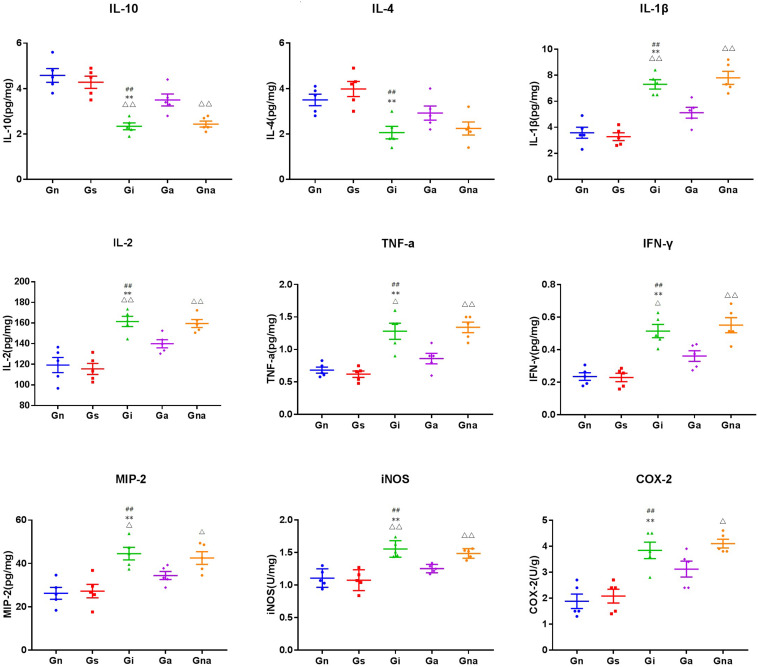
Effect of acupuncture on the levels of related inflammatory mediators in the hippocampus. The data are expressed as the mean ± SEM (*n* = 5). **P < 0.01, vs. Gn group; ^#^*P* < 0.05, ^##^*P* < 0.01, vs. Gs group; ^△^*P* < 0.05, ^△△^*P* < 0.01, vs. Ga group. Gn, normal group; Gs, sham-operated group; Gi, impaired group; Ga, acupoint group; Gna, non-acupoint group.

The levels of cytokines (i.e., IL-1β, IL-2, IL-4, IL-10, TNF-α, and INF-γ), chemokines (i.e., MIP-2), and other inflammatory mediators (i.e., COX-2 and iNOS) in the hippocampus were detected by the ELISA kits (Figure 7). There were no significant differences in all indexes between the Gs and Gn groups (*P* > 0.05), excluding the effect of the operation on the level of hippocampal inflammation in rats. Compared with the Gn and Gs groups, the levels of cytokines, such as IL-1β, IL-2, TNF-α, INF-γ, MIP-2, COX-2, and iNOS, in the hippocampus in the Gi group were increased, while IL-4 and IL-10 were decreased (all *P* < 0.01), which were similar to the results of serum cytokines. It further suggested that there was inflammation in VD rats. However, compared with the Gi group, the hippocampal proinflammatory factors in the Ga group IL-1β (*P* < 0.01), IL-2 (*P* < 0.01), TNF-α (*P* < 0.05), INF-γ (*P* < 0.05), MIP-2 (*P* < 0.05), iNOS (*P* < 0.01), and COX-2 were decreased after acupuncture treatment, whereas the anti-inflammatory factors IL-4 and IL-10 (*P* < 0.01) were increased, indicating that acupuncture could improve the inflammation in the hippocampus of VD rats. This effect was not observed in the Gna group compared with the Gi group (*P* > 0.05), indicating the specificity of acupuncture.

## Discussion

Currently, there is no specific drug for VD (Sun, 2018), as a treatment option, acupuncture shows its potential in the treatment of VD (Liu et al., 2014). In this study, we confirmed that acupuncture treatment could improve the cognitive impairment of the VD rats. Moreover, we also found that there are abnormalities of peripheral inflammation and immune function in the VD rats, and acupuncture can regulate this abnormality.

Specifically, on the one hand, we found that the proportion of T cells and CD4+ helper T cells in the peripheral blood and spleen of the Gi group decreased in varying degrees, accompanied by the decrease in the ratio of CD4/CD8, which means that there is an abnormal immune function in the VD rats. T lymphocyte plays a key role in cell-mediated adaptive immunity. Lots of evidence supported the involvement of T lymphocytes in neuroinflammation and brain injury after ischemia (An et al., 2014). The abnormal function of CD4 + T cells may lead to serious cognitive impairment (Jeon et al., 2016), and some clinical evidence (Busse et al., 2017) has also proven that the proportion of T cells and CD4+ helper T cells decreased in the peripheral blood of patients with VD. Combined with our results, we speculated that adaptive immunity may be involved in the pathological process of cognitive impairment after cerebral ischemia, especially T cells and CD4+ helper T cells. However, after the acupuncture treatment, the proportion of T cells and CD4+ helper T cells increased to a certain extent, and the cognitive impairment was improved. This indicates that the therapeutic effect of acupuncture may be related to T cells and CD4+ helper T cells. Some animal studies have also shown that T cells and CD4+ helper T cells contribute to nerve repair and neurogenesis (Brait et al., 2012; Wang et al., 2017). Another interesting finding is that in the spleen of model rats, the proportion of B cells increased significantly, whereas the increase in peripheral blood was not obvious. This phenomenon seems to suggest that B lymphocytes are involved in the process of cognitive impairment after multiple infarctions. Some evidence also shows that although B lymphocytes have a certain protective effect in the early stage of cerebral ischemia, the autoimmunity mediated by B lymphocytes may be an important mechanism of cognitive impairment after stroke (Doyle Kristian and Buckwalter Marion, 2017). However, after acupuncture, the proportion of B cells did not change. A reasonable explanation is that the therapeutic effect of acupuncture may not be mediated by B cells, or acupuncture may not change the proportion of B cells but may change its subsets or functions, which needs further research. On the other hand, we found that there was inflammation in the peripheral blood of model rats, which showed the upregulation of inflammatory mediators such as IL-1β, IL-2, TNF-α, INF-γ, MIP-2, COX-2, and iNOS and the downregulation of anti-inflammatory factors such as IL-4 and IL-10, which was consistent with the results of some studies. Some data showed that IL-1β, IL-2, IL-6, TNF-α, INF-γ, and other inflammatory factors in peripheral blood of patients with VD increased in varying degrees (Guoping et al., 2015; Schmitz et al., 2015), while IL-4 and IL-10 decreased in varying degrees (De Luigi et al., 2002; Liu et al., 2018). A large number of data show that peripheral inflammation can cause neuroinflammation and cognitive impairment (Badshah et al., 2016; Miryam and Goar, 2016; Zhang et al., 2018) and can aggravate the symptoms of VD model rats (Levine et al., 2015). After acupuncture, inflammatory mediators such as IL-1β, IL-2, TNF-α, INF-γ, MIP-2, COX-2, iNOS, and other inflammatory mediators were downregulated in varying degrees, while IL-4 and IL-10 were upregulated. This indicates that acupuncture reduced the level of peripheral inflammation, improved the peripheral immune environment, and reversed peripheral inflammation and the corrected peripheral immune abnormalities can reduce neuroinflammation and cognitive impairment (Dadsetan et al., 2016; Balzano et al., 2020). This may be one of the mechanisms of acupuncture improving the cognitive function of VD model rats.

Finally, in addition to improving peripheral immune function and inflammation, we also found that acupuncture can improve the inflammation level of the hippocampus. The results of ELISA indicate that there was inflammation in the hippocampus, and the level of inflammation was alleviated after acupuncture treatment.

## Conclusion

Our results found that the VD rats have an abnormal immune function and peripheral inflammation, while acupuncture can regulate the immune cell phenotype and peripheral inflammation of VD rats, reduce the expression of proinflammatory factors in the hippocampus, and improve the cognitive impairment of VD rats. This shows that acupuncture can be used as an efficacious therapeutic approach for VD in the future.

## Data Availability Statement

The raw data supporting the conclusions of this article will be made available by the authors, without undue reservation.

## Ethics Statement

The animal study was reviewed and approved by the guidelines of the Committee for Animal Care at Tianjin University of Traditional Chinese Medicine.

## Author Contributions

PP and XZ contributed to the conception and design of the study. ZM and ZZ organized the database. ZL and YW performed the statistical analysis. PP and ZM wrote the first draft of the manuscript. QL, XL, PX, DY, HZ, and RW wrote sections of the manuscript. All the authors participated in and completed the whole experiment, contributed to manuscript revision, read, and approved the submitted version.

## Conflict of Interest

The authors declare that the research was conducted in the absence of any commercial or financial relationships that could be construed as a potential conflict of interest.

## References

[B1] An C. Shi Y. Li P. Hu X. Gan Y. Stetler R. A. (2014). Molecular dialogs between the ischemic brain and the peripheral immune system: dualistic roles in injury and repair[J]. *Prog. Neurobiol.* 115 6–24. 10.1016/j.pneurobio.2013.12.002 24374228 PMC4014303

[B2] Badshah H. Ali T. Kim M. O. (2016). Osmotin attenuates LPS-induced neuroinflammation and memory impairments via the TLR4/NFκB signaling pathway[J]. *Sci. Rep.* 6:24493. 10.1038/srep24493 PMC4837357 27093924

[B3] Balzano T. Dadsetan S. Forteza J. Cabrera-Pastor A. Taoro-Gonzalez L. Malaguarnera M. (2020). Chronic hyperammonemia induces peripheral inflammation that leads to cognitive impairment in rats: reversed by anti-TNF-α treatment[J]. *J. Hepatol.* 73 582–592. 10.1016/j.jhep.2019.01.008 30654069

[B4] Brait V. H. Arumugam T. V. Drummond G. R. Sobey C. G. (2012). Importance of T lymphocytes in brain injury, immunodeficiency, and recovery after cerebral ischemia[J]. *J. Cereb. Blood FlowMetab.* 32 598–611. 10.1038/jcbfm.2012.6 22293986 PMC3318155

[B5] Busse M. Michler E. von Hoff F. Dobrowolny H. Hartig R. Frodl T. (2017). Alterations in the peripheral immune system in dementia [J]. *J. Alzheimers Dis. JAD* 58:1303. 10.3233/jad-161304 28582858

[B6] Cai R. L. Cheng H. L. Zhou T. Chen G. Q. Chen X. S. Wu S. B. (2015). Effects of electroacupuncture on learning-memory ability and expression of hippocampal vascular endothelial growth factor (VEGF), VEGF receptor 1 and 2 genes in vascular cognitive impairment rats[J]. *Acupunct. Res.* 40 25–29. 25845216

[B7] Chen L. Xu A. Yin N. Zhao M. Wang Z. Chen T. (2017). Enhancement of immune cytokines and splenic CD4+ T cells by electroacupuncture at ST36 acupoint of SD rats[J]. *PLoS One* 12:e0175568. 10.1371/journal.pone.0175568 28406959 PMC5391063

[B8] Cho K. O. Kim S. K. Kim S. Y. (2017). Chronic cerebral hypoperfusion and plasticity of the posterior cerebral artery following permanent bilateral common carotid artery occlusion[J]. *Korean J. Physiol. Pharmacol.* 21 643–650. 10.4196/kjpp.2017.21.6.643 29200907 PMC5709481

[B9] Dadsetan S. Balzano T. Forteza J. Cabrera-Pastor A. Taoro-Gonzalez L. Hernandez-Rabaza V. (2016). Reducing peripheral inflammation with infliximab reduces neuroinflammation and improves cognition in rats with hepatic encephalopathy[J]. *Front. Mol. Neurosci.* 9:106. 10.3389/fnmol.2016.00106 27853420 PMC5089983

[B10] De Luigi A. Pizzimenti S. Quadri P. Lucca U. Tettamanti M. Fragiacomo C. (2002). Peripheral inflammatory response in alzheimer’s disease and multiinfarct dementia[J]. *Neurobiol. Dis.* 11 308–314. 10.1006/nbdi.2002.0556 12505423

[B11] Dionisio-Santos D. A. Olschowka J. A. O’Banion M. K. (2019). Exploiting microglial and peripheral immune cell crosstalk to treat Alzheimer’s disease[J]. *J. Neuroinflammation* 16:74. 10.1186/s12974-019-1453-0 PMC6449993 30953557

[B12] Doyle K. P. Quach L. N. Solé M. Axtell R. C. Nguyen T. V. Soler-Llavina G. J. (2015). B-Lymphocyte-mediated delayed cognitive impairment following Stroke[J]. *J. Neurosci.* 35 2133–2145. 10.1523/jneurosci.4098-14.2015 25653369 PMC4315838

[B13] Doyle Kristian P. Buckwalter Marion S. (2017). Does B lymphocyte-mediated autoimmunity contribute to post-stroke dementia?[J]. *Brain Behav. Immun.* 64 1–8. 10.1016/j.bbi.2016.08.009 27531189 PMC5305803

[B14] Edrissi H. Schock S. C. Cadonic R. Hakim A. M. Thompson C. S. (2016). Cilostazol reduces blood brain barrier dysfunction, white matter lesion formation and motor deficits following chronic cerebral hypoperfusion[J]. *Brain Res.* 1646 494–503. 10.1016/j.brainres.2016.06.036 27350079

[B15] Fang Y. Sui R. (2016). Electroacupuncture at the wangu acupoint suppresses expression of inflammatory cytokines in the hippocampus of rats with vascular dementia[J]. *Afr. J. Tradit. Comp. Altern. Med.* 13 17–24. 10.21010/ajtcam.v13i5.3 PMC5416636 28487889

[B16] Guoping P. Wei W. Xiaoyan L. Fangping H. Zhongqin C. Benyan L. (2015). Characteristics of the peripheral T cell immune response of patients at different stages of vascular cognitive impairment[J]. *Immunol. Lett.* 168 120–125. 10.1016/j.imlet.2015.09.015 26433058

[B17] Harland M. Torres S. Liu J. Wang X. (2020). Neuronal mitochondria modulation of LPS-induced neuroinflammation[J]. *J Neurosci* 40 1756–1765. 10.1523/jneurosci.2324-19.2020 31937559 PMC7046320

[B18] Hei Y. Chen R. Yi X. Long Q. Gao D. Liu W. (2018). HMGB1 neutralization attenuates hippocampal neuronal death and cognitive impairment in rats with chronic cerebral hypoperfusion via suppressing inflammatory responses and oxidative stress[J]. *Neuroscience* 383 150–159. 10.1016/j.neuroscience.2018.05.010 29777754

[B19] Iadecola C. (2010). The overlap between neurodegenerative and vascular factors in the pathogenesis of dementia[J]. *Acta Neuropathol.* 120 287–296. 10.1007/s00401-010-0718-6 20623294 PMC3001188

[B20] Impellizzeri D. Siracusa R. Cordaro M. Crupi R. Peritore A. F. Gugliandolo E. (2019). N-Palmitoylethanolamine-oxazoline (PEA- OXA): a new therapeutic strategy to reduce neuroinflammation, oxidative stress associated to vascular dementia in an experimental model of repeated bilateral common carotid arteries occlusion[J]. *Neurobiol. Dis.* 125 77–91. 10.1016/j.nbd.2019.01.007 30660740

[B21] Jeon S. G. Kim K. A. Chung H. Choi J. Song E. J. Han S. Y. (2016). Impaired memory in ot-II transgenic mice is associated with decreased adult hippocampal neurogenesis possibly induced by alteration in th2 cytokine Levels[J]. *Mol. Cells* 39 603–610. 10.14348/molcells.2016.0072 27432189 PMC4990752

[B22] Kipnis J. Cohen H. Cardon M. Ziv Y. Schwartz M. (2004). T cell deficiency leads to cognitive dysfunction: implications for therapeutic vaccination for schizophrenia and other psychiatric conditions[J]. *Proc. Natl. Acad. Sci. U.S.A.* 101 8180–8185. 10.1073/pnas.0402268101 15141078 PMC419577

[B23] Levine D. A. Galecki A. T. Langa K. M. Unverzagt F. W. Kabeto M. U. Giordani B. (2015). Trajectory of cognitive decline after incident stroke[J]. *J. Am. Med. Assoc.* 314:41. 10.1001/jama.2015.6968 26151265 PMC4655087

[B24] Liu F. Li Z. M. Jiang Y. J. Chen L. D. (2014). A meta-analysis of acupuncture use in the treatment of cognitive impairment after stroke[J]. *J. Altern. Comp. Med.* 20 535–544. 10.1089/acm.2013.0364 24915606 PMC4086349

[B25] Liu Q. Q. Zhong D. Zhang X. Li G. Z. (2018). IL-10 targets Th1/Th2 balance in vascular dementia[J]. *Eur. Rev. Med. Pharmacol. Sci.* 22 5614–5619. 30229836 10.26355/eurrev_201809_15826

[B26] Miryam N. C. Goar G. (2016). LPS-induced murine neuroinflammation model: main features and suitability for pre-clinical assessment of nutraceuticals[J]. *Curr. Neuropharmacol.* 14 155–164. 10.2174/1570159x14666151204122017 26639457 PMC4825946

[B27] Mori M. A. Meyer E. Soares L. M. Milani H. Guimarães F. S. de Oliveira R. M. W. (2017). Cannabidiol reduces neuroinflammation and promotes neuroplasticity and functional recovery after brain ischemia[J]. *Prog. Neuro Psychopharmacol. Biol. Psychiatry* 75 94–105. 10.1016/j.pnpbp.2016.11.005 27889412

[B28] Nyitrai G. Spisák T. Spisák Z. Gajári D. Diószegi P. Kincses T. Z. (2018). Stepwise occlusion of the carotid arteries of the rat: MRI assessment of the effect of donepezil and hypoperfusion-induced brain atrophy and white matter microstructural changes[J]. *PLoS One* 13:e0198265. 10.1371/journal.pone.0198265 29851990 PMC5979036

[B29] O’Brien J. T. Thomas A. (2015). Vascular dementia[J]. *Lancet* 386 1698–1706. 26595643 10.1016/S0140-6736(15)00463-8

[B30] Peng W. N. Zhao H. Liu Z. S. Wang S. (2007). Acupuncture for vascular dementia[J]. *Cochrane Database Syst. Rev.* 2:CD004987. 10.1002/14651858.CD004987.pub2 PMC9022668 17443563

[B31] Plassman B. L. Langa K. M. Fisher G. G. Heeringa S. G. Weir D. R. Ofstedal M. B. (2007). Prevalence of dementia in the United States: the aging, demographics, and memory study[J]. *Neuroepidemiology* 29 125–132. 10.1159/000109998 17975326 PMC2705925

[B32] Prinz M. Priller J. (2017). The role of peripheral immune cells in the CNS in steady state and disease[J]. *Nat. Neurosci.* 20 136–144. 10.1038/nn.4475 28092660

[B33] Ranjithkumar R. Alhadidi Q. Shah Z. A. Ramanathan M. (2019). Tribulusterine containing tribulus terrestris extract exhibited neuroprotection through attenuating stress kinases mediated inflammatory mechanism: in vitro and in vivo studies[J]. *Neurochem. Res.* 44 1228–1242. 10.1007/s11064-019-02768-7 30863969

[B34] Schmitz M. Hermann P. Oikonomou P. Stoeck K. Ebert E. Poliakova T. (2015). Cytokine profiles and the role of cellular prion protein in patients with vascular dementia and vascular encephalopathy[J]. *Neurobiol. Aging* 36 2597–2606. 10.1016/j.neurobiolaging.2015.05.013 26170132

[B35] Shang J. Yamashita T. Fukui Y. Song D. Li X. Zhai Y. (2018). Different associations of plasma biomarkers in alzheimer’s disease, mild cognitive impairment, vascular dementia, and ischemic stroke[J]. *J. Clin. Neurol.* 14 29–34. 10.3988/jcn.2018.14.1.29 29629537 PMC5765253

[B36] Shi G. X. Liu C. Z. Li Q. Q. Zhu H. Wang L. P. (2012). Influence of acupuncture on cognitive function and markers of oxidative DNA damage in patients with vascular dementia[J]. *J. Tradit. Chin. Med.* 32 199–202. 10.1016/s0254-6272(13)60011-4 22876443

[B37] Smith E. E. (2017). Clinical presentations and epidemiology of vascular dementia[J]. *Clin. Sci.* 131 1059–1068. 10.1042/cs20160607 28515342

[B38] Sun M. K. (2018). Potential therapeutics for vascular cognitive impairment and dementia[J]. *Curr. Neuropharmacol.* 16 1036–1044. 10.2174/1570159x15666171016164734 29046153 PMC6120112

[B39] Venkat P. Chopp M. Chen J. (2015). Models and mechanisms of vascular dementia[J]. *Exp. Neurol.* 272 97–108. 10.1016/j.expneurol.2015.05.006 25987538 PMC4631710

[B40] Wan F. Niu X. Song Y. L. Si Y. C. (2016). The role of chinese herbs and acupuncture on the inflammation reaction after cerebral ischemia[J]. *Curr. Pharm. Design* 22 709–719. 10.2174/1381612822666151204001348 26635265

[B41] Wang G. Y. Taylor T. Sumich A. Merien F. Borotkanics R. Wrapson W. (2017). Associations between immunological function and memory recall in healthy adults[J]. *Brain Cogn.* 119 39–44. 10.1016/j.bandc.2017.10.002 29020639

[B42] Wang X. R. Shi G. X. Yang J. W. Yan C. Q. Lin L. T. Du S. Q. (2015). Acupuncture ameliorates cognitive impairment and hippocampus neuronal loss in experimental vascular dementia through Nrf2-mediated antioxidant response[J]. *Free Radical Biol. Med.* 89 1077–1084. 10.1016/j.freeradbiomed.2015.10.426 26546103

[B43] Yu J. Zhang X. Liu C. Meng Y. Han J. (2006). Effect of acupuncture treatment on vascular dementia[J]. *Neurol. Res.* 28 97–103. 10.1179/016164106x91951 16464371

[B44] Yuan-Cheng K. Li Z. Ying S. Li Y. Ren W. L. Wei W. S. (2018). MicroRNA-26b regulates the microglial inflammatory response in hypoxia/ischemia and affects the development of vascular cognitive impairment[J]. *Front. Cell. Neurosci.* 12:154. 10.3389/fncel.2018.00154 29937716 PMC6002499

[B45] Zakaria R. Wan Yaacob W. M. Othman Z. Long I. Ahmad A. H. Al-Rahbi B. (2017). Lipopolysaccharide-induced memory impairment in rats: a model of Alzheimer’s disease[J]. *Physiol. Res.* 66 553–565. 10.33549/physiolres.933480 28406691

[B46] Zhang D. Xiao Y. Lv P. Teng Z. Dong Y. Qi Q. (2017). Edaravone attenuates oxidative stress induced by chronic cerebral hypoperfusion injury: role of ERK/Nrf2/HO-1 signaling pathway[J]. *Neurol. Res.* 40 1–10. 10.1080/01616412.2017.1376457 29125058

[B47] Zhang F. Zhang J. G. Yang W. Xu P. Xiao Y. L. Zhang H. T. (2018). 6-Gingerol attenuates LPS-induced neuroinflammation and cognitive impairment partially via suppressing astrocyte overactivation[J]. *Biomed. Pharmacother.* 107 1523–1529. 10.1016/j.biopha.2018.08.136 30257370

[B48] Zhang Z. H. Shi G. X. Li Q. Q. Wang Y. J. Li P. Zhao J. X. (2014). Comparison of cognitive performance between two rat models of vascular dementia[J]. *Int. J. Neurosci.* 124 818–823. 10.3109/00207454.2014.880435 24397495

[B49] Zhao C. Bao C. Li J. Zhu Y. Wang S. Yang L. (2015). Moxibustion and acupuncture ameliorate crohn’s disease by regulating the balance between Th17 and Treg cells in the intestinal mucosa[J]. *Evid. Based Comp. Altern. Med.* 2015:938054. 10.1155/2015/938054 PMC4539447 26347488

[B50] Zhao L. Shen P. Han Y. Zhang X. Nie K. Cheng H. (2011). Effects of acupuncture on glycometabolic enzymes in multi-infarct dementia rats[J]. *Neurochem. Res.* 36 693–700. 10.1007/s11064-010-0378-x 21279683

